# 832. Body Mass Index and Quality of Life in People Living With HIV

**DOI:** 10.1093/ofid/ofab466.1028

**Published:** 2021-12-04

**Authors:** Jennifer Ken-Opurum, Girish Prajapati, Joana E Matos, Swarnali Goswami, Princy N Kumar

**Affiliations:** 1 Kantar Health, New York, New York; 2 Merck & Co., Inc., Rahway, NJ; 3 Merck & Co., Inc., Kenilworth, NJ (Author was working under an internship in partnership with The University of Mississippi, University, MS), University, Mississippi; 4 Georgetown University School of Medicine, Washington, District of Columbia

## Abstract

**Background:**

Weight gain among people living with HIV (PLWH) on antiretroviral therapy (ART) may lead to obesity. This study evaluated association between body mass index (BMI) and health-related quality of life (HRQoL) from the patient’s perspective.

**Methods:**

A cross-sectional study using self-reported data from the 2018 and 2019 US National Health and Wellness Survey (NHWS), a nationally representative online survey of ~75,000 adults was conducted. Respondents self-reporting a physician diagnosis of and prescription use for treatment of HIV were included. HRQoL was assessed using Short-Form 36-Item Health Survey Version 2 [Mental and Physical Component Summary scores (MCS and PCS)] and EQ-5D-5L [dimension responses: “no” vs “any problems”/“yes”); EQ-Visual Analogue Scale (VAS) score]. Bivariate analyses (chi-square tests for categorical and ANOVA for continuous variables) compared patient characteristics and HRQoL outcomes across BMI (kg/m^2^) categories: normal weight (NW; 18.5-< 25), overweight (OW; 25-< 30) and obese (OB; ≥30). Multivariable models analyzed each outcome as a function of BMI, controlling for age, sex, race, and Charlson Comorbidity Index (CCI; excluding HIV/AIDS).

**Results:**

A total of 566 respondents were analyzed. Majority were aged ≥50 years (58%) and male (87%). The OB (vs NW) group had higher proportion of respondents who were female (22% vs 10%), Black (37% vs 24%), residing in the South (46% vs 33%), and higher mean CCI score (1.28 vs. 0.97) (Table 1). A higher proportion of OB (vs NW) respondents reported having pain/discomfort and problems with mobility and usual activities but not self-care. Anxiety/depression was reported less in OB vs NW groups (Table 1) However, self-reported use of prescription medications for anxiety (19% vs 20%) and depression (34% vs 25%) was similar in OB and NW groups. PCS and EQ-VAS scores were lower in OB vs OW and NW, but no difference in MCS score was observed (Table 1). Lower PCS and EQ-VAS scores were associated with higher BMI (both p=0.01) but not MCS (p=0.68) in multivariate models.

**Conclusion:**

PLWH with higher BMI have poorer physical and general HRQoL. Impact of potential adverse weight gain and transition to higher BMI on humanistic and clinical outcomes should be considered when selecting ART regimens.

Table 1. Comorbidity Burden and Quality of Life in People Living with HIV by BMI Categories.

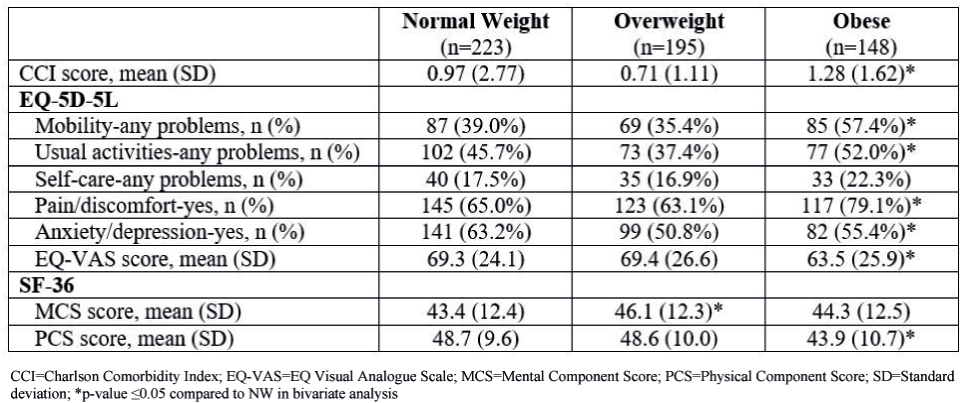

**Disclosures:**

**Jennifer Ken-Opurum, PhD**, **Kantar Health** (Employee) **Girish Prajapati, M.B.B.S., MPH** , **Merck & Co., Inc.** (Employee, Shareholder) **Joana E. Matos, PhD**, **Kantar Health** (Employee) **Princy N. Kumar, MD**, **AMGEN** (Other Financial or Material Support, Honoraria)**Eli Lilly** (Grant/Research Support)**Gilead** (Grant/Research Support, Shareholder, Other Financial or Material Support, Honoraria)**GSK** (Grant/Research Support, Shareholder, Other Financial or Material Support, Honoraria)**Merck & Co., Inc.** (Grant/Research Support, Shareholder, Other Financial or Material Support, Honoraria)

